# Carbon dioxide combining power as a predictor of unplanned ICU transfer in hospital wards: a retrospective cohort study

**DOI:** 10.3389/fmed.2025.1724891

**Published:** 2025-12-11

**Authors:** Li Li, Luo Yang, Zhao Xin, Du Yao, Hu Xianchun, Wu Moxin

**Affiliations:** 1 Department of Intensive Care Unit, International Peace Maternity and Child Health Hospital, School of Medicine, Shanghai Jiao Tong University, Shanghai, China; 2 Shanghai Key Laboratory of Embryo Original Diseases, Shanghai, China; 3 Department of Intensive Care Unit, Jiujiang University Affiliated Hospital, Jiujiang, China; 4 Department of Oncology, Jiujiang Third People’s Hospital, Jiujiang, China; 5 Department of Clinical Medicine, Jiujiang University Affiliated Hospital, Jiujiang, China

**Keywords:** carbon dioxide combining power, hospital wards, intensive care unit, mortality, predictive value, retrospective cohort study

## Abstract

**Background:**

Unplanned intensive care unit (ICU) transfers are associated with high mortality. Conventional early warning scores like modified Early Warning Score (MEWS) show limited predictive ability. Carbon dioxide combining power (CO2CP), a routine acid-base parameter, may enable earlier detection of clinical deterioration.

**Objective:**

To evaluate CO2CP as a predictor of mortality in unplanned ICU transfers and assess its additive value to traditional scoring systems.

**Methods:**

This single-center retrospective study analyzed 101 adults with unplanned ICU transfers (Feb 2024-Jun 2025). CO2CP levels within 8 h pre-transfer, MEWS, and APACHE II scores were collected. Primary outcome was ICU mortality. Analyses included ROC curves, survival analysis, multivariable logistic regression, decision curve analysis, and correlation assessment.

**Results:**

The area under the ROC curve (AUC) for CO2CP was 0.722 (95% CI: 0.615–0.829), higher than that of MEWS (AUC = 0.528, *p* = 0.040). Bootstrap validation yielded an AUC of 0.723 (95% CI: 0.581–0.849), indicating relative stability. Survival analysis showed poorer outcomes in patients with lower CO2CP (log-rank *P* = 0.003). In multivariable analysis, CO2CP remained independently associated with mortality (OR ≈ 0.87 per 1 mmol/L increase, *p* < 0.001), although the effect size was modest. The combined CO2CP-MEWS model demonstrated good discriminatory ability, although its clinical utility requires further confirmation.

**Conclusion:**

Carbon dioxide combining power may serve as an independent predictor of mortality risk in unplanned ICU transfers. Its integration with MEWS may enhance early risk stratification in general wards, though these findings require validation in larger prospective studies.

## 1 Introduction

Unplanned transfer occurs when patients are moved from general wards to the intensive care unit (ICU) due to unexpected clinical decline. This definition excludes planned transfers, such as those following surgery or related to medical procedures (1). Studies indicate that patients with unplanned ICU transfer have significantly higher mortality rates and longer hospital stays than those admitted directly to the ICU (2). The risk is particularly elevated for those transferred more than 48 h after admission. In this group, the mortality rate increases 19-fold, and the duration of hospitalization doubles (3). A key reason for unplanned ICU transfers is the failure of ward staff to recognize early signs of clinical deterioration promptly. This underscores an urgent need for early, simple, and reliable risk stratification tools.

Although early warning scores (EWS), such as the National Early Warning Score (NEWS) and the Modified Early Warning Score (MEWS), have been incorporated into routine ward practice, their ability to promptly identify deteriorating patients remains very limited (4). Torvik MA et al. (5) report that NEWS flags fewer than one-third of patients who will need unplanned ICU transfer, yet over one-third whose condition worsens still score below the medium-high threshold. Meanwhile, 18.4% of ward in-patients record at least one NEWS ≥5, but the vast majority never reach the ICU. Similarly, Murujosaet al. (6) point out that a considerable proportion (67%) of patients with unplanned ICU transfer had a NEWS score that did not trigger a risk warning prior to transfer. The EWS primarily assesses patients’ acute risk by monitoring macroscopic physiological indicators such as vital signs and level of consciousness. However, it neglects the earlier occurring acid-base disturbances within the body.

Carbon dioxide combining power (CO2CP), which reflects the bicarbonate content in plasma, serves as a key indicator of the body’s alkaline reserve and acid–base status. Although direct arterial blood gas (ABG) analysis has largely replaced CO2CP for evaluating acid–base imbalance, obtaining arterial samples remains impractical in general wards. CO2CP therefore retains clinical value, especially for detecting acid–base disturbances in non-respiratory patients. Therefore, this study aimed to: (1) evaluate the predictive value of CO2CP for ICU mortality in patients with unplanned ICU transfers; and (2) assess whether the combination of CO2CP and MEWS provides additive predictive value compared to MEWS alone.

## 2 Materials and methods

### 2.1 Study design and participants

This study employed a single-center retrospective cohort design and included adult patients who experienced unplanned ICU transfer between February 2024 and June 2025. Inclusion criteria were as follows: ➀ age ≥ 18 years; ➁ direct transfer from a general ward to the ICU, excluding transfers from the emergency department, operating room, or other departments; ➂ at least one CO2CP test completed within 8 h before ICU admission (during general ward treatment); ➃ survival in the ICU for ≥24 h to ensure valid outcome assessment; ➄ complete electronic medical records, including baseline demographic information, laboratory test results, scoring system records, and prognostic data. Exclusion criteria included: ➀ pregnant or breastfeeding women; ➁ patients with terminal cancer or receiving palliative care; ➂ patients with acute exacerbations of chronic obstructive pulmonary disease or chronic renal failure requiring long-term dialysis; ➃ patients who voluntarily discharged or transferred out of the hospital for personal reasons, making complete prognostic information unavailable; ➄ patients transferred directly from the emergency department or operating room to the ICU; ➅ patients with missing key data ≥20%, affecting the accuracy of statistical analysis. After screening 537 cases, 101 patients met the criteria and were included in the final analysis.

### 2.2 Data collection

Data were extracted from the hospital electronic medical record system (EMR) and included the following categories: ➀ Baseline information, such as age and gender; ➁ Laboratory indicators, including CO2CP levels measured within 8 h prior to ICU transfer, along with other relevant inflammatory markers such as white blood cell count (WBC); ➂ Scoring system data, including MEWS at the time of ICU admission and acute physiology and chronic health evaluation II (APACHE II) within 24 h after admission. MEWS was calculated based on heart rate, systolic blood pressure, respiratory rate, body temperature, and level of consciousness at the time of transfer, while APACHE II was calculated using the worst physiological parameters (e.g., arterial blood pressure, heart rate, respiratory rate) and chronic health status (e.g., presence of chronic organ dysfunction) within 24 h after admission; ➃ Outcome indicators, with all-cause mortality during ICU stay as the primary outcome, confirmed through discharge diagnoses, death records, or follow-up information in the EMR.

### 2.3 Ethical considerations

The study protocol strictly adhered to the ethical guidelines of the Declaration of Helsinki and was approved by the Institutional Review Board (IRB) of Jiujiang University Affiliated Hospital (approval number: No.jjuhmeer-b 2023-10-1). Given that this study was a retrospective observational study involving only secondary analysis of anonymized clinical data that had been prospectively collected, with no additional patient interventions and no potential risk to patient privacy or rights, the IRB granted a waiver for patient consent. All data access and processing followed strict confidentiality protocols, with patient personal information de-identified to ensure privacy and security.

### 2.4 Statistical methods

All statistical analyses in this study were conducted using GraphPad Prism 10.0 and R 4.5.0 software, with a significance level set at α = 0.05 (two-sided). ➀ Continuous variables were expressed as mean ± standard deviation ($\bar{\text{x}}\pm \text{s}$), and categorical variables were described in terms of frequency (percentage). ➁ The predictive performance of CO2CP, MEWS, and APACHE II was evaluated using ROC curves and internal validation through Boots set resampling (*n* = 5000). ➂ Decision curve analysis (DCA) was further applied to calculate and plot the net benefit curves across various threshold probabilities, thereby quantifying the clinical utility of the CO2CP model alone and in combination with the MEWS model. ➃ Kaplan-Meier survival curves were generated and compared using the log-rank test. ➄ Multivariable logistic regression was used to identify independent predictors of mortality, with results expressed as Odds Ratios (OR) and their corresponding 95% Confidence Intervals (CI). ➅ Finally, the linear relationships between CO2CP and acid-base parameters (BE, lactate) were examined using Pearson correlation analysis, to comprehensively validate the predictive efficacy, clinical benefit, and physiological basis of CO2CP.

## 3 Results

### 3.1 Baseline characteristics

A total of 101 patients were included, with an ICU mortality rate of 23.8% (24/101). Patients were divided into survivor (*n* = 77) and non-survivor (*n* = 24) groups. There were no significant differences between the groups in terms of age (65.62 ± 2.194 years in survivors vs. 69.25 ± 3.497 years in non-survivors, *P* = 0.8431), gender (54.55% male in survivors vs. 60.87% in non-survivors, *P* = 0.9901), or prevalence of comorbidities such as hypertension, diabetes, and coronary heart disease (all *P* > 0.05). The only significant difference was in the cause of ICU admission, with a higher proportion of septic shock in the non-survivor group (45.8%) compared to the survivor group (31.2%, *P* = 0.006) (Table 1). After Welch-corrected t-tests and Benjamini-Hochberg FDR correction of 12 laboratory indicators, 3 indicators (CO2CP, APACHE II, WBC) were considered to have exploratory significant differences (*q* < 0.05) (Table 2).

**TABLE 1:** Comparing the baseline characteristics of study patients with unplanned ICU transfers between the non-survivor and survivor groups.

Variable	Total sample (*n* = 101)	Survivor group (*n* = 77)	Non-survivor group (*n* = 24)	*P*-value
**Age (years)**	66.49 ± 1.886	65.62 ± 2.194	69.25 ± 3.497	0.8431
**Male (*n*, %)**	56 (56.0%)	42 (54.55%)	14 (60.87%)	0.9901
**Comorbidities (n, %)**				
**Hypertension**	53 (52.5%)	37 (48.1%)	16 (66.7%)	0.1108
**Diabetes**	38 (37.6%)	27 (35.1%)	11 (45.8%)	0.3474
**Coronary heart disease**	48 (47.5%)	35 (45.5%)	13 (54.2%)	0.4904
**Cause of ICU admission (n, %)**				
**Septic shock**	35 (34.7%)	24 (31.2%)	11 (45.8%)	0.006
**Hemorrhagic shock**	13 (12.9%)	10 (13.0%)	3 (12.5%)	0.754
**Severe pneumonia**	12 (11.9%)	9 (11.7%)	3 (12.5%)	0.725
**Heart failure**	11 (10.9%)	10 (13.0%)	1 (4.2%)	0.083
**Acute renal failure**	10 (9.9%)	9 (11.7%)	1 (4.2%)	0.122
**Others**	20 (19.8%)	15 (19.5%)	5 (20.8%)	0.754

Data are presented as mean ± standard deviation (SD) or percentage.

**TABLE 2:** Comparison of baseline laboratory indicators between the non-survivor and survivor groups (Welch *t*-test + FDR correction).

Variable	Non-survivors (*n* = 24)	Survivors (*n* = 77)	Difference (95% CI)	t	df	*p*	q
**CO2CP (mmol/L)**	14.05 ± 8.48	20.44 ± 5.59	−6.39 (−9.9 to −2.9)	3.47	29.5	0.002	0.012
**Apache ii (score)**	34.75 ± 8.78	26.51 ± 7.10	8.24 (4.4–12.1)	4.19	32.9	<0.001	0.003
**Mews (score)**	6.96 ± 4.10	5.91 ± 2.03	1.05 (−0.7 to 2.8)	1.21	26.6	0.238	0.330
**WBC (×10^9^/L)**	18.50 ± 8.16	12.52 ± 7.01	5.98 (2.4–9.6)	3.24	34.3	0.003	0.012
**BNP (pg/mL)**	11.360 ± 12.278	6.040 ± 8.923	5.320 (−1.80 to 10.820)	1.97	30.9	0.058	0.163
**Alb (g/L)**	30.04 ± 5.60	30.93 ± 5.77	−0.89 (−3.5 to 1.7)	0.68	39.5	0.503	0.587
**Cr (μmol/L)**	338.1 ± 341.1	242.4 ± 324.9	95.7 (−62 to 254)	1.21	36.9	0.233	0.330
**CRP (mg/L)**	115.4 ± 136.6	125.4 ± 118.8	−10.0 (−72 to 52)	0.32	34.5	0.749	0.749
**HB (g/L)**	106.4 ± 26.7	96.9 ± 28.1	9.5 (−4.1 to 23.1)	1.51	40.2	0.140	0.320
**RBC (×10^12^/L)**	3.51 ± 0.87	3.27 ± 0.89	0.23 (−0.18 to 0.64)	1.15	39.2	0.259	0.330
**PLT (×10^9^/L)**	184.7 ± 117.7	149.8 ± 77.0	34.9 (−16 to 86)	1.37	29.4	0.183	0.320
**D-Dimer (U/L)**	5 160 ± 10 051	4 105 ± 6 531	1 055 (−3300 to 5410)	0.48	29.3	0.632	0.681

q = Benjamini-Hochberg adjusted p; exploratory significance defined as *q* < 0.05. BNP (pg/mL), B-type Natriuretic Peptide; Alb (g/L), Albumin; Cr (μmol/L), Creatinine; CRP (mg/L), C-reactive Protein; Hb (g/L), Hemoglobin; RBC (×10^12^/L), Red Blood Cell; Hct (%), Hematocrit; WBC (×10^9^/L), White Blood Cell; PLT (×10^9^/L), Platelet.

### 3.2 The predictive efficacy of single indicators (ROC curve analysis) and internal validation through Boots set resampling (*n* = 5000)

**FIGURE 1:**
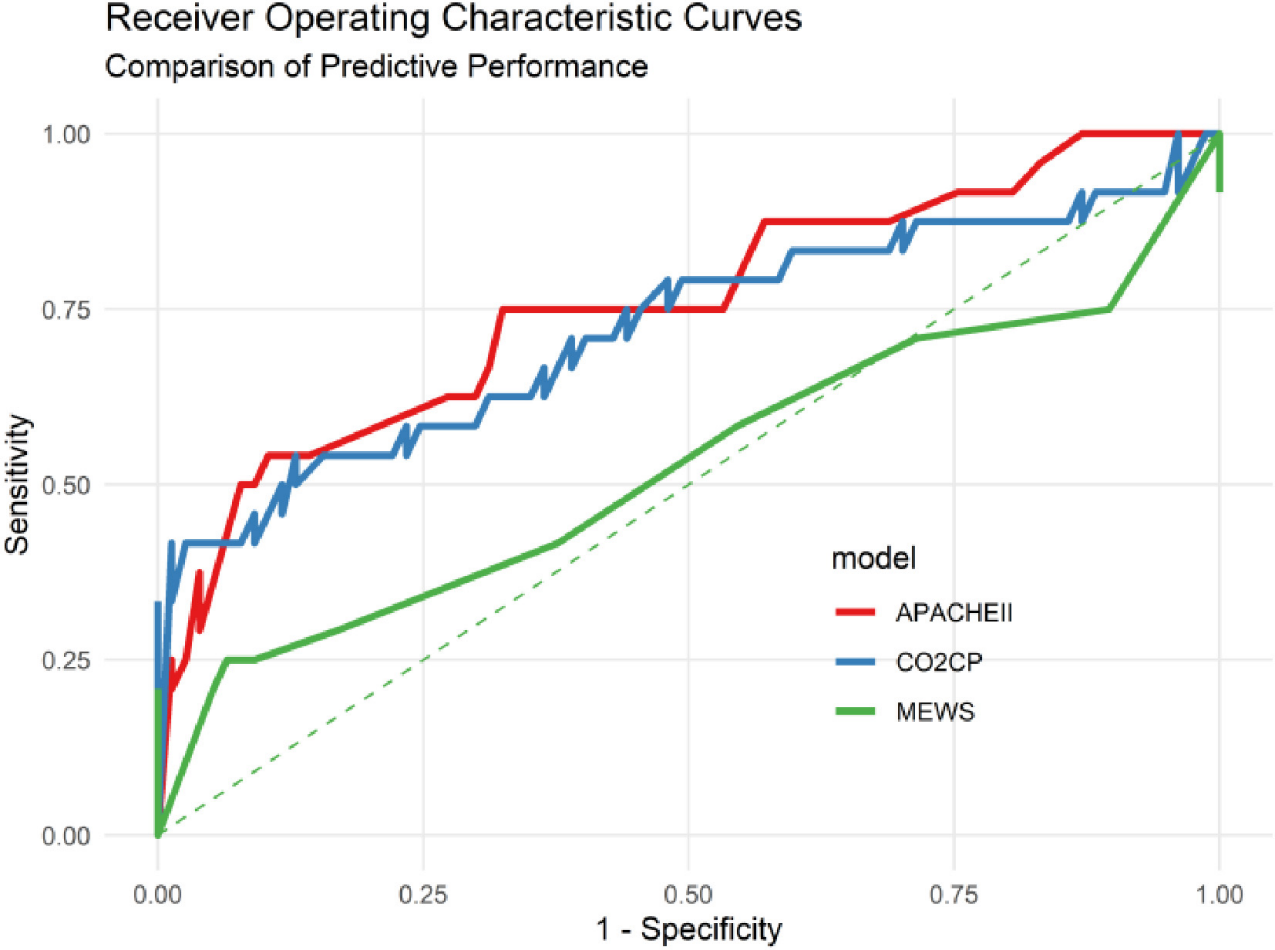
Receiver operating characteristic (ROC) curves of CO2CP, MEWS, and APACHE II for predicting mortality in patients with unplanned ICU transfer. The red curve represents the APACHE II score; the blue curve represents CO2CP; the green curve represents the MEWS score.

**FIGURE 2:**
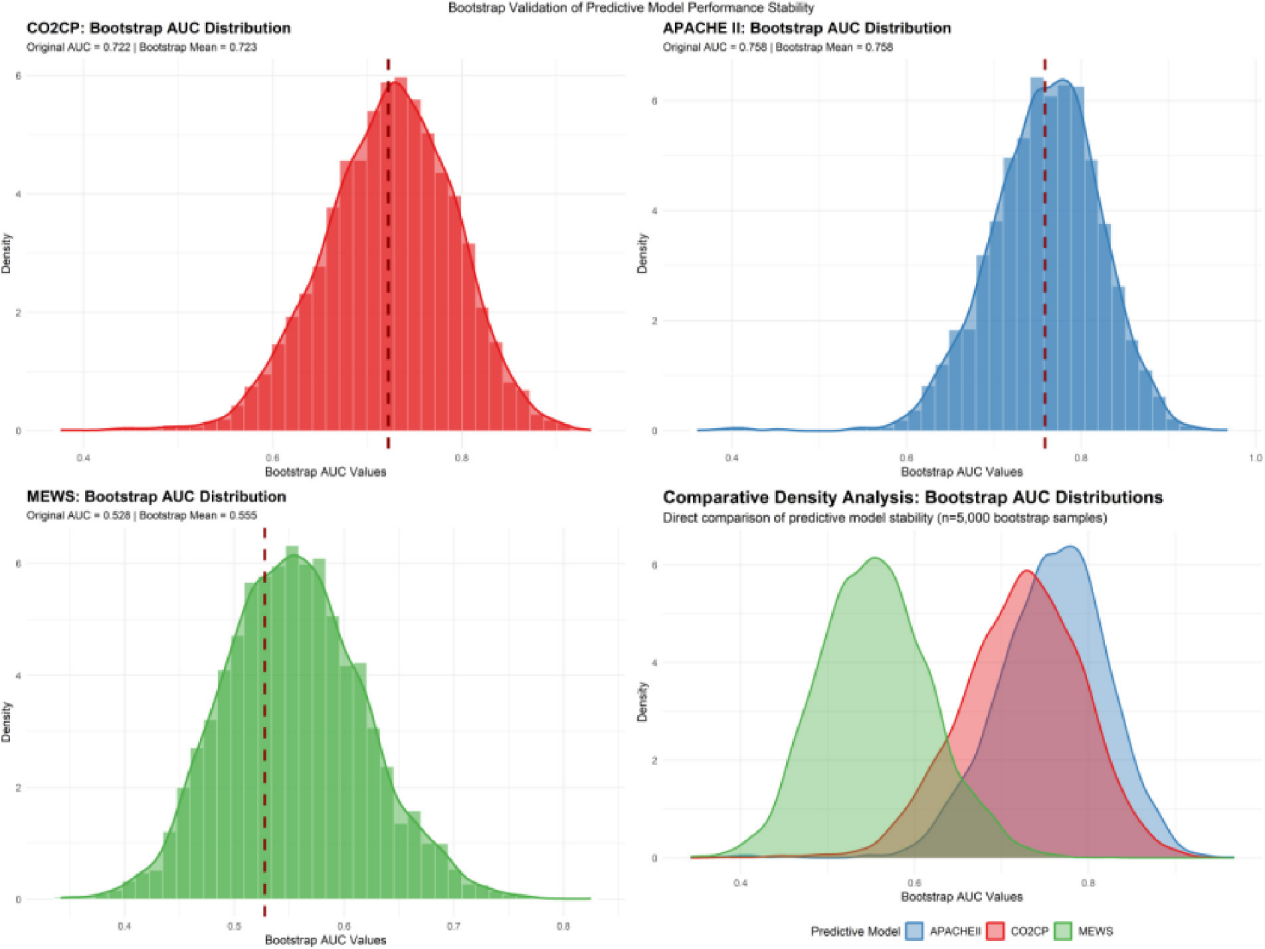
Bootstrap resampling was performed 5000 times to calculate the confidence interval of AUC. Red line, CO2CP; blue line, APACHE II score; green line, MEWS score.

To evaluate the discriminative performance and stability of various predictive indicators, this study employed receiver operating characteristic (ROC) curve analysis to compare the predictive efficacy of CO2CP, MEWS, and APACHE II, and conducted internal validation through Bootstrap resampling (*n* = 5000). The ROC analysis showed that the area under the curve (AUC) for predicting ICU mortality risk by CO2CP was 0.722 (95% CI: 0.585–0.860), which was significantly better than that of MEWS (AUC = 0.528, 95% CI: 0.372–0.683; DeLong test *P* = 0.0396) and had no statistical difference compared with APACHE II (AUC = 0.758, 95% CI: 0.637–0.879; DeLong test *P* = 0.6195). For details of the comparison of ROC curves of each index, (see Figure 1 and Table 3). To verify the stability of the model, Bootstrap resampling was performed 5000 times to calculate the confidence interval of AUC. The Bootstrap AUC for CO2CP was 0.723 (95% CI: 0.581–0.849), for APACHE II it was 0.758 (95% CI: 0.630–0.874), and for MEWS it was 0.555 (95% CI: 0.438–0.687), which were highly consistent with the original results. The Bootstrap distribution chart (see Figure 2 and Table 3) showed that the AUC values of each index were concentrated, indicating that the results were robust.

**TABLE 3:** Comparison of the predictive performance of various indicators for mortality risk (DeLong method, *n* = 101).

Predictive indicator	AUC (95% CI)	vs. MEWS (*P*-value)	vs. APACHE II (*P*-value)	Bootstrap mean ± SD (95% CI)
**MEWS**	0.528 (0.372–0.683)	–	–	0.555 ± 0.070 (0.438–0.687)
**APACHE II**	0.758 (0.637–0.879)	0.0007	–	0.758 ± 0.064 (0.630–0.874)
**CO2CP**	0.722 (0.585–0.860)	0.0396	0.6195	0.723 ± 0.064 (0.581–0.849)

AUC, area under the receiver operating characteristic curve; CI, confidence interval. *P*-values are derived from pairwise comparisons of ROC curves (DeLong’s test).

### 3.3 Exploration of clinical utility via decision curve analysis

**FIGURE 3:**
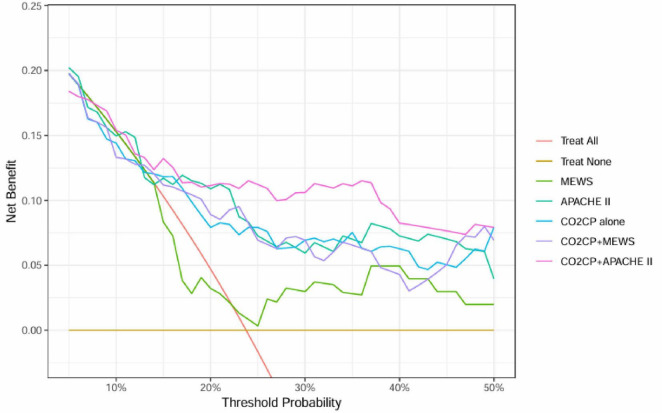
Decision curve analysis (DCA) of different prediction models for ICU mortality risk. The *x*-axis represents the threshold probability, and the *y*-axis represents the net benefit. The curves include “Treat All” (red line), “Treat None” (orange line), MEWS (green line), APACHE II (cyan line), CO2CP (blue line), CO2CP + MEWS (purple line), and CO2CP + APACHE II (pink line).

Given the limited sample size of this study (*n* = 101, with only 24 mortality events), the stability of indicators such as NRI and IDI is still uncertain. Therefore, we focused on employing decision curve analysis (DCA) to preliminarily explore the potential clinical utility of CO2CP, both alone and in combination with traditional scoring systems (MEWS, APACHE II), for mortality risk prediction. The DCA results, which reflect the net benefit of each model across different decision thresholds, are shown in Figure 3.

In the single-indicator models, the net benefit curve for MEWS remained close to the “Treat None” line, indicating limited clinical utility. The curves for APACHE II and CO2CP were comparable, both demonstrating a moderate net benefit over a range of threshold probabilities, suggesting potential clinical value. In the combined models, the integration of MEWS and CO2CP showed a wider range of threshold probabilities with improved net benefit compared to MEWS alone, and even outperformed other models in certain ranges. This suggests that the MEWS + CO2CP combination may hold promise for clinical application in ward settings. Conversely, combining CO2CP with APACHE II did not yield a substantial improvement in net benefit over APACHE II alone.

### 3.4 Survival analysis

**FIGURE 4:**
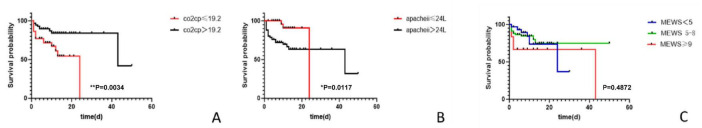
Kaplan–Meier survival curves stratified by CO2CP (A), APACHE II (B), and MEWS (C) levels. Differences between curves were evaluated with the log-rank (Mantel-Cox) and Gehan-Breslow-Wilcoxon tests. (A) CO2CP: log-rank χ^2^ = 8.578, df = 1, *P* = 0.0034; Gehan-Breslow χ^2^ = 6.055, df = 1, *P* = 0.0139. (B) APACHE II: log-rank χ^2^ = 6.352, df = 1, *P* = 0.0117; Gehan-Breslow χ^2^ = 9.784, df = 1, *P* = 0.0018. (C) MEWS: log-rank χ^2^ = 1.438, df = 2, *P* = 0.4872; Gehan-Breslow χ^2^ = 2.656, df = 2, *P* = 0.2651. Significance thresholds: *P < 0.05, **P < 0.01, ns = not significant.

Kaplan-Meier analysis with log-rank testing revealed significant prognostic disparities among the evaluated indices. Patients stratified by CO2CP cut-off 19.2 mmol/L showed divergent survival curves (Log-rank *P* = 0.0034), with the low CO2CP group having a significantly reduced median survival of 24.0 days compared to 43.0 days in the high CO2CP group (Mantel-Haenszel HR = 3.534, 95% CI: 1.518–8.225) (see Figure 4A). Similarly, survival outcomes stratified by APACHE II score cut-off 24 also differed (Log-rank *P* = 0.0117; HR = 3.990, 95% CI: 1.756–9.065) (see Figure 4B). In contrast, stratification by MEWS score (MEWS < 5, 5 ≤ MEWS ≤ 8, MEWS ≥ 9) failed to significantly differentiate survival risks (Log-rank *P* = 0.4872) (see Figure 4C). These preliminary findings suggest that CO2CP and APACHE II may serve as potential prognostic markers for unplanned ICU transfers. However, due to the limited sample size (*n* = 101, 24 events), cautious interpretation and further validation are needed.

### 3.5 Multivariable logistic regression analysis

After multivariable logistic regression analysis (101 complete cases), CO2CP remained the only independent predictor of in-hospital mortality after adjustment for MEWS, age and sex: each 1 mmol/L increase in CO2CP was associated with a 13% reduction in the odds of death (OR 0.87, 95% CI 0.80–0.94, *P* < 0.001). In contrast, MEWS, age and sex did not achieve statistical significance (P ≥ 0.191). The model demonstrated good discrimination (AUROC 0.733, 95% CI 0.61–0.86, *P* < 0.001) and calibration (Hosmer–Lemeshow χ^2^ = 14.51, *P* = 0.070), with an overall classification accuracy of 85.2% at the 0.5 probability threshold (see Table 4).

**TABLE 4:** Multivariable logistic regression of in-hospital mortality (*n* = 101, complete cases).

Variable	β	SE	OR	95% CI	*P*
**Intercept**	−0.63	1.40	0.53	0.03 – 7.74	0.653
**CO2CP (per 1 mmol/L)**	−0.14	0.04	0.87	0.80 – 0.94	<0.001
**MEWS (per 1 point)**	0.07	0.10	1.07	0.89 – 1.30	0.485
**Age (per 1 year)**	0.02	0.02	1.02	0.99 – 1.06	0.191
**Sex (male vs. female)**	0.18	0.54	1.20	0.42 – 3.55	0.734

### 3.6 Correlation and consistency analysis

**FIGURE 5:**
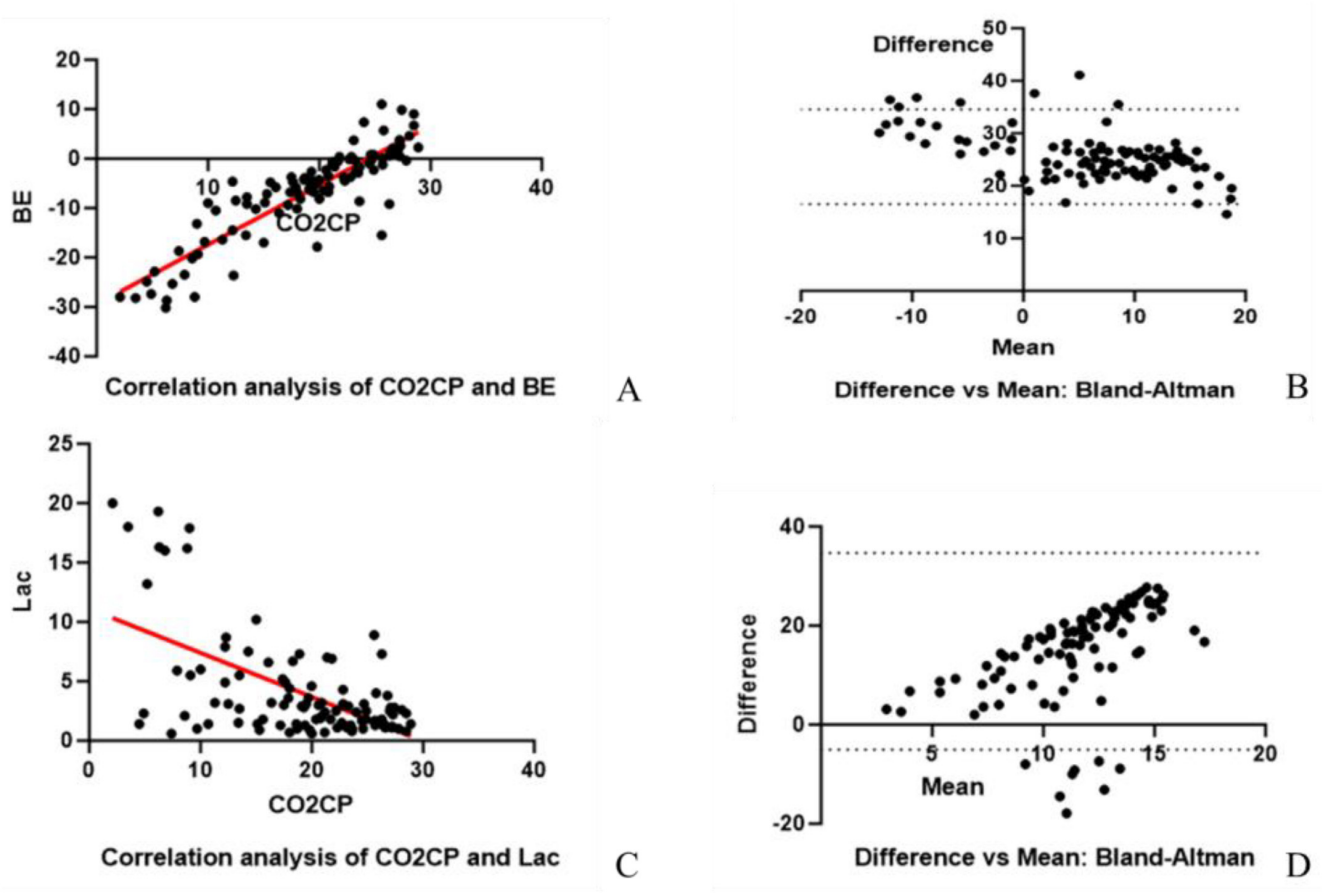
Linear correlation analysis. (A) CO2CP is highly correlated with BE (R^2^ = 0.78, *P* < 0.001), with the dashed lines indicating the 95% confidence band; Sy.x = 4.4 mmol/L; (B) Bland-Altman plot: The mean bias is 25.6 mmol/L, and the 95% limits of agreement fall within the physiologically acceptable range; (C) CO2CP is moderately negatively correlated with lactate (*r* = –0.58, R^2^ = 0.34); (D) Bland-Altman plot: The limits of agreement are ± 20 mmol/L, indicating that CO2CP should not be used to estimate lactate levels.

Linear regression showed a strong linear correlation between CO2CP and base excess (BE) (R^2^ = 0.78, *P* < 0.001, Figure 5A), with a standard estimation error Sy.x = 4.4 mmol/L, indicating a reliable trend in the population, but individual estimation still needs to refer to blood gas results (Figure 5B). The Bland-Altman results showed a systematic bias of 25.6 mmol/L between the two methods, and the 95% limits of agreement fell within the physiologically expected range, which did not affect the clinical application of CO2CP as an independent indicator. CO2CP was moderately negatively correlated with lactate (*r* = −0.58, R^2^ = 0.34, Figure 5C), with wide limits of agreement ± 20 mmol/L (Figure 5D), and CO2CP could not replace lactate measurement at the bedside.

## 4 Discussion

This study represents the first preliminary exploration of the prognostic value of CO2CP, a routine laboratory parameter, in patients experiencing unplanned ICU transfer. Our study yielded two main findings, which still need further validation. First, compared with MEWS alone, CO2CP demonstrated stronger discriminative ability for predicting mortality. Second, the combined model generated by integrating CO2CP with MEWS showed enhanced predictive performance. Survival analysis and multivariate logistic regression analysis preliminarily revealed the independent association of CO2CP.

Carbon dioxide combining power is a fundamental, rapid, and routinely available test parameter that reflects the body’s bicarbonate buffering capacity and metabolic acid–base status. The role of CO2CP in this study may stem from its sensitivity to acid–base disturbances, particularly metabolic acidosis, which is a common pathway in critically ill patients due to hypoperfusion (e.g., septic shock), renal dysfunction, or other metabolic derangements. Our findings align with the growing evidence highlighting the value of acid–base balance in prognosis. For instance, He et al. (7) identified CO2CP as an independent predictor of sepsis-related liver injury, while Hu et al. (8) reported that decreased CO2CP contributes to acute kidney injury and mortality in non-respiratory patients. Similarly, in neurology, low CO2CP has been associated with poor short-term prognosis after acute ischemic stroke (9, 10). Our study extends this concept to the critical period of clinical deterioration on general wards prior to ICU transfer, positioning CO2CP as a pragmatic parameter for early risk stratification.

Although ABG analysis remains the gold standard for precise acid–base evaluation, and serum lactate levels (11–13) and BE (14, 15) have been widely recognized for their value in predicting mortality in critically ill patients, the procurement of ABG is invasive, requires specialized skills, and carries a non-negligible risk of serious complications, as emphasized by Rowling et al. (16). Moreover, the correct interpretation of ABG can be challenging for ward staff (17, 18). In contrast, CO2CP can be rapidly measured on venous blood, is inexpensive, and is already a part of routine clinical laboratory tests. This makes it a feasible and scalable tool for serial monitoring in general wards. Our correlation analysis supports the physiological rationale for its use. We found a strong linear correlation between CO2CP and BE (R^2^ = 0.78, *p* < 0.001), a primary acid–base parameter from ABG. The Bland-Altman analysis, although revealing a systematic bias, showed that the 95% limits of agreement fell within a physiologically acceptable range, indicating that CO2CP trends reliably reflect BE trends and can be used for the initial assessment of the body’s acid–base status.

Our study cautiously explored a well-known limitation of conventional EWS (such as MEWS), which rely on macroscopic vital signs and level of consciousness and neglect acid–base disturbances that have already occurred within the body, resulting in an inability to identify deteriorating patients in a timely and accurate manner (19). The introduction of CO2CP effectively compensates for this deficiency by providing an objective parameter that can immediately capture potential acid–base disturbances. This approach of enhancing EWS with laboratory parameters is gaining attention. For example, Ünal Çetin et al. (20) improved mortality prediction in sepsis by combining MEWS with the lactate/albumin ratio, while other studies have combined lactate with NEWS and the Predisposition, Infection, Response, and Organ Dysfunction (PIRO) scores (21). In general wards, combining MEWS with routine laboratory indicators can also enhance the predictive value of mortality (22). However, nurse compliance with routine MEWS assessments is already low (23, 24), and complex combined prediction models may further reduce compliance rates and hinder the timely identification of critically ill patients. The “MEWS + CO2CP” model, with its simplicity and immediacy, is more practical in general wards. Decision curve analysis in this study showed that the combined model may have higher net benefit across a wide range of threshold probabilities, but this finding needs to be further validated in studies with larger sample sizes.

Several limitations of our study must be acknowledged. First, the single-center retrospective design may limit the generalizability of our findings and introduce the possibility of unmeasured confounding. Second, the limited sample size (*n* = 101) may reduce estimate precision. Finally, although standardized, the definition of unplanned ICU transfer retains some subjectivity; nevertheless, 5,000-fold Bootstrap resampling indicated that the findings are internally stable. Future research should focus on external validation in a multicenter, prospective cohort with a larger and more diverse patient population. In addition, intervention studies are needed to assess whether the implementation of this combined alert system can actually reduce the number of unplanned ICU transfers and mortality.

## 5 Conclusion

This study suggests that CO2CP may serve as a potential predictor of mortality risk in patients with unplanned ICU transfer; its combination with MEWS may help identify high-risk patients in general wards. The aforementioned results require external validation in larger-scale, prospective, multicenter cohorts and further assessment of its actual clinical benefits through interventional studies.

## Data Availability

The original contributions presented in this study are included in this article/supplementary material, further inquiries can be directed to the corresponding author.
